# Synthesis and structures of three isoxazole-containing Schiff bases

**DOI:** 10.1107/S2053229620010530

**Published:** 2020-08-29

**Authors:** Helen E. Mason, Judith A. K. Howard, Hazel A. Sparkes

**Affiliations:** a Department of Chemistry, Durham University, South Road, Durham DH1 3LE, England; b School of Chemistry, University of Bristol, Cantock’s Close, Bristol BS8 1TS, England

**Keywords:** Schiff base, chromism, isoxazole, phenol, crystal structure, hydrogen bonding

## Abstract

The synthesis and structures of three isoxazole-containing Schiff bases are reported, namely, (*E*)-2-{[(isoxazol-3-yl)imino]­meth­yl}phenol, C_10_H_8_N_2_O_2_, (*E*)-2-{[(5-methyl­isoxazol-3-yl)imino]­meth­yl}phenol, C_11_H_10_N_2_O_2_, and (*E*)-2,4-di-*tert*-butyl-6-{[(isoxazol-3-yl)imino]­meth­yl}phenol, C_18_H_24_N_2_O_2_. All three structures contain an intra­molecular O—H⋯N hydrogen bond, alongside weaker inter­molecular C—H⋯N and C—H⋯O contacts. The C—O(H) and imine C=N bond lengths were consistent with structures existing in the enol rather than the keto form. Despite having dihedral angles <25°, none of the com­pounds were observed to be strongly thermochromic, unlike their anil counterparts; however, all three com­pounds showed a visible colour change upon irradiation with UV light.

## Abstract 2

Three {[(isoxazol-3-yl)imino]­meth­yl}phenols were synthesized and structurally characterized. All three structures contain an intra­molecular O—H⋯N hydrogen bond and none were found to be strongly thermochromic.

## Introduction   

A wide range of Schiff bases can be relatively easily prepared making them versatile as ligands and consequently they have found widespread use over many years in areas such as organometallic chemistry (Kargar *et al.*, 2020 ▸), polymer synthesis (Mighani, 2020 ▸), anti­cancer drugs (Parveen, 2020 ▸), catalysts (Kumari *et al.*, 2019 ▸) and sensors (Sahu *et al.*, 2020 ▸). In addition, Schiff bases themselves have been found to display inter­esting properties with anils, *i.e.* Schiff bases of salicyl­aldehyde derivatives with aniline derivatives, having been first found to exhibit both thermo- and photochromism in the solid state (Senier *et al.*, 1909 ▸; Cohen & Schmidt, 1962 ▸; Cohen *et al.*, 1964 ▸). Originally, the thermo- and photochromism of anils were thought to be mutually exclusive (Cohen & Schmidt, 1962 ▸; Cohen *et al.*, 1964 ▸), but this has since been found not to be the case and it is thought they all display thermochromism with some also displaying photochromism (Fujiwara *et al.*, 2004 ▸). The colour change is believed to be due to a photo- or thermally induced tautomeric equilibrium shift between colourless enol(–imine) and keto(–amine) forms (Hadjoudis & Mavridis, 2004 ▸; Robert *et al.*, 2009 ▸).

The Schiff bases of salicyl­aldehyde (2-hy­droxy­benz­al­de­hyde) derivatives with isoxazole derivatives have not been widely characterized structurally, with a search of the Cam­bridge Structural Database (CSD; Version of June 2020; Groom *et al.*, 2016 ▸) revealing two structures, namely, (*E*)-2-meth­oxy-6-{[(5-methyl­isoxazol-3-yl)imino]­meth­yl}phenol (refcode GITGIA; Zhao *et al.*, 2008 ▸) and *N*-(5-methyl­isoxazol-3-yl)-3,5-di-*tert*-butyl­salicyl­aldimine (refcode YINFAD; Çelik *et al.*, 2007 ▸). Herein the synthesis and characterization of three isoxazole-con­taining Schiff bases are reported, namely, (*E*)-2-{[(isoxazol-3-yl)imino]­meth­yl}phenol, **1**, (*E*)-2-{[(5-methyl­isoxazol-3-yl)imino]­meth­yl}phenol, **2**, and (*E*)-2,4-di-*tert*-butyl-6-{[(isoxazol-3-yl)imino]­meth­yl}phenol, **3** (see Scheme 1).

## Experimental   

### Synthesis   

All reagents were used as supplied by Aldrich. Compounds were synthesized by direct condensation of the appropriate salicyl­aldehyde and isoxazole derivatives in ethanol. The salicyl­aldehyde (0.0025 mol) and aniline (0.0025 mol) were each dissolved in ethanol (25 ml). The resulting solutions were combined and refluxed with stirring for 6–8 h. Any precipitate was filtered off, rinsed with ethanol and left to dry. The (remaining) solution was then rotary evaporated until (further) precipitate formed. Recrystallization was carried out from hexa­ne–di­chloro­methane for **1**, ethanol for **2** or chloro­form for **3** (see Scheme 1).

### Characterization   

Elemental C, H and N content analysis was carried out using the Durham University Analytical service on an Exeter Analytical E-440 Elemental Analyzer. Mass spectrometry in positive electrospray (ES+) mode was performed by the Durham University Mass Spectrometry service on a Waters TQD with an Acquity solvent system. Full details are available in the supporting information.

### Refinement   

All H atoms, apart from the hy­droxy H atom involved in intra­molecular hydrogen bonding with the imine N atom, were positioned geometrically and refined using a riding model. The H atoms involved in the intra­molecular hydrogen bonding were located in a Fourier difference map wherever feasible. 
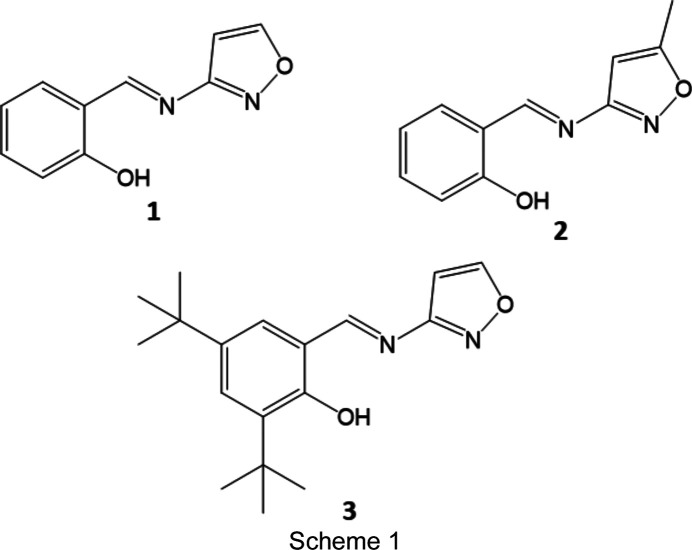
Compounds **1** and **2** crystallized in noncentrosymmetric space groups; however, the Flack parameters obtained were not meaningful as the data were collected with molybdenum radiation and there are no heavy atoms to facilitate anomalous dispersion. In **3**, which contained two independent mol­ecules in the asymmetric unit, one of the *tert*-butyl groups was disordered; the sum of the occupancies of the two parts was set to equal 1 and subsequently fixed at the refined values. The inter­planar dihedral angle was calculated by measuring the angle between planes computed through the five or six non-H atoms of the two rings. See Table 1 ▸ for further details of the crystallographic data collections.

## Results and discussion   

### Structural discussion   

**Figure 1:**
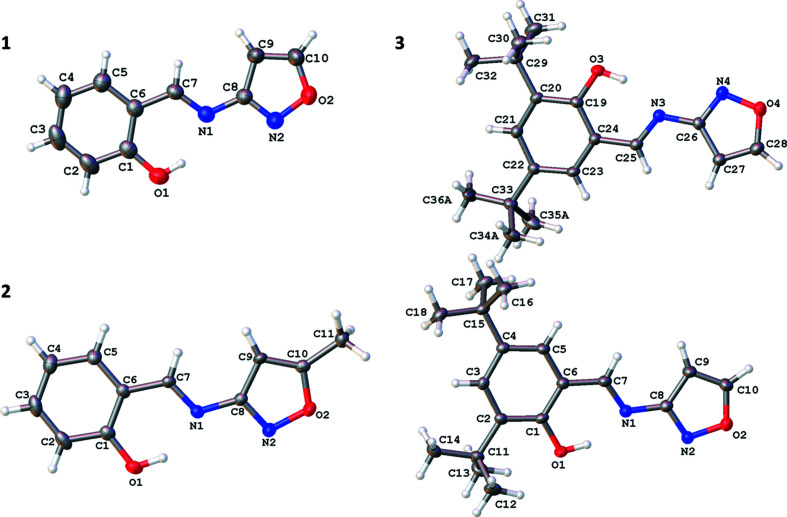
Illustration of the structures of **1** [at 210 (2) K], **2** [120 (2) K] and **3** [120 (2) K], with the atomic numbering schemes depicted. Anisotropic displacement parameters are drawn at the 50% probability level. In the case of **3**, only one position of the disordered *tert*-butyl group is shown for clarity.

The structures of **1**–**3** all consist of the same basic backbone with a hy­droxy-substituted arene group joined to an isoxazole ring *via* an imine (C=N) group (Fig. 1 ▸). The C7=N1 bond lengths are consistent with the presence of a double bond [ranging from 1.283 (2) Å in **1** to 1.293 (2) Å in **3**], while the C1—O1 bond lengths [ranging from 1.350 (2) Å in **1** to 1.3655 (18) Å in **3**] are consistent with a single bond. Indeed, the hy­droxy H atom was located in a Fourier difference map in the vicinity of the O atom, supporting the fact that the structures are all in the more commonly observed enol form rather than the keto form. All three structures contain an intra­molecular O1—H1⋯N1 hydrogen bond with similar parameters, *e.g.* the O1⋯N1 distances range from 2.6062 (17) to 2.632 (2) Å (Tables 2 ▸–4 ▸ ▸). The structures also contain weaker inter­molecular C—H⋯N and C—H⋯O inter­actions (Tables 2 ▸–4 ▸ ▸).

**Figure 2:**
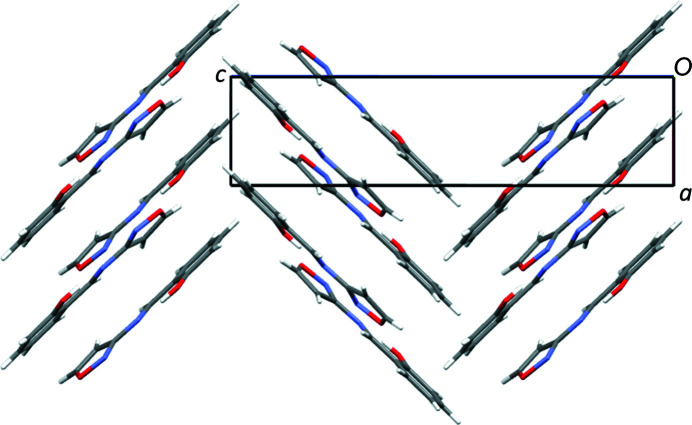
Illustration of the packing in **1**, looking down the *b* axis.

Examining the structure of **1**, short π–π stacking type inter­actions are found between the six-membered aromatic ring and the C=N group [centroid-to-centroid distance = 3.2905 (3) Å] (Corne *et al.*, 2016 ▸), creating one-dimensional stacks in approximately the [101] direction. The inter­molecular inter­actions involving the isoxazole N atom and the OH group are: (i) bifurcated C—H⋯N inter­actions to other mol­ecules; (ii) bifurcated C—H⋯O inter­actions to two different mol­ecules. These inter­actions link a central mol­ecule with four mol­ecules in total, *i.e.* two mol­ecules either side of itself, creating chains in approximately the *b*-axis direction. Combining these inter­actions with the π–π stacking creates a three-dimensional network with a herringbone-type packing structure (Fig. 2 ▸).

**Figure 3:**
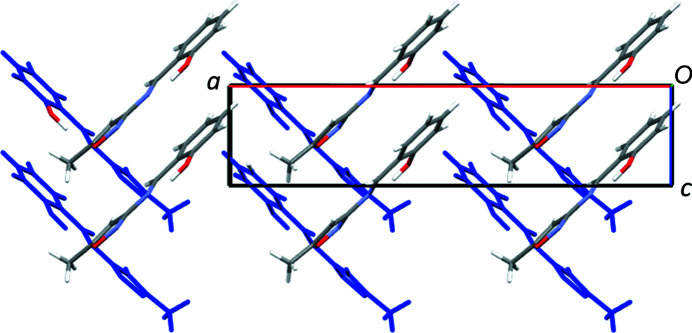
Illustration of the packing in **2**, looking down the *b* axis. Mol­ecules are shown in elemental colours (C grey, O red, N blue and H white) at the front, while mol­ecules shown in blue are one mol­ecule down the *b* axis, showing the different orientations.

The structure of **2** has short π–π stacking type inter­actions that exist between the six-membered aromatic ring and the C=N group [centroid-to-centroid distance = 3.2772 (1) Å], creating a one-dimensional stack approximately up the [101] direction. All the stacks in the *ac* plane are in the same direction; however, moving in the *b*-axis direction by one mol­ecule, the stacks in the *ac* plane are in different directions due to the presence of the 2_1_ screw axes and glide planes. The structure also contains: (i) C—H⋯N and C—H⋯O inter­actions involving the N and O atoms of isoxazole; (ii) C—H⋯O inter­actions involving the O atom of the OH group. These inter­actions link the central mol­ecule to four others, two on each side of the mol­ecule, creating a three-dimensional network. An illustration of the overall packing is shown in Fig. 3 ▸.

**Figure 4:**
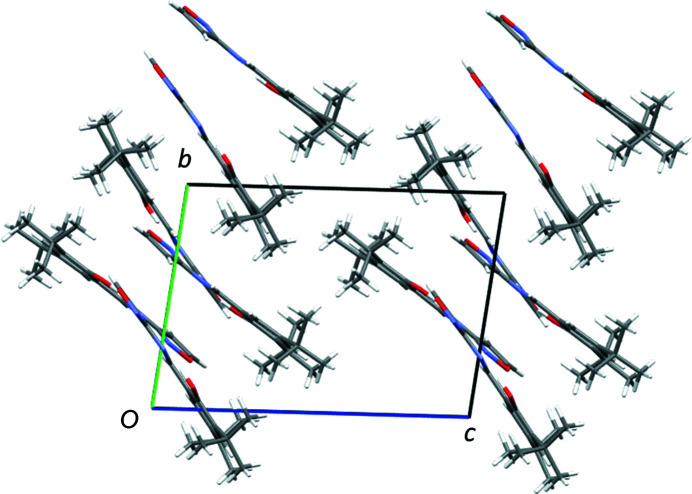
Illustration of the packing in **3**, looking down the *a* axis.

In **3**, the two independent mol­ecules show slightly different inter­molecular inter­actions: (i) C—H⋯N (bifurcated for the isoxazole ring containing atoms N2 and O2, and not for the isoxazole ring containing atoms N4 and O4) and a C—H⋯O inter­action involving the N and O atoms of isoxazole; (ii) C—H⋯O inter­actions involving the O atom of the OH group. This creates a three-dimensional packing network (Fig. 4 ▸). There are no π–π stacking type inter­actions between the six-membered aromatic ring and the C=N group in this case, presumably because of the presence of the bulky *tert*-butyl groups.

### Chromic studies   

The chromic behaviour of com­pounds **1**–**3** was not fully investigated herein; however, some observations are worth reporting given the similarity of the structures to the widely studied anils. Schiff bases of salicyl­aldehyde derivatives with aniline derivatives, which exhibit both thermo- and photochromism in the solid state (Cohen & Schmidt, 1962 ▸; Cohen *et al.*, 1964 ▸; Fujiwara *et al.*, 2004 ▸). In anils, a link has been proposed between the dihedral angle (Φ) and the chromic behaviour of some of the Schiff bases, with a suggestion that com­pounds with Φ < 25° are expected to be strongly thermochromic, while those with Φ > 25° are more likely to be photochromic (Hadjoudis & Mavridis, 2004 ▸; Robert *et al.*, 2009 ▸). Clearly the dihedral angle is not the only factor that has been found to influence chromism in anils, with thermochromic structures tending to be more closely packed than photochromic structures and substituents that weaken the O—H bond or strengthen the accepting ability of the N atom often resulting in more strongly thermochromic complexes (Hadjoudis & Mavridis, 2004 ▸; Robert *et al.*, 2009 ▸). The Schiff bases of salicyl­aldehyde derivatives with isoxazole derivatives presented here have not been widely studied in terms of their chromic behaviour and the three com­pounds presented herein appear to show some differences from the anils. The Φ value was 6.95 (12)° for **1**, 4.42 (14)° for **2** and 6.53 (10)/14.27 (8)° (two molecules) for **3**; however, none of the com­pounds were observed to be strongly thermochromic by eye when cooled to ∼80 K. In the case of **2** and **3**, this is perhaps not a major surprise as they are yellow at room temperature and, while they did become paler in colour at lower temperatures, the strongly thermochromic anil com­pounds are typically a red/orange colour at room temperature and change to yellow upon cooling. However, **1**, which is orange at room temperature, remained an orange colour at ∼80 K also. All three com­pounds did show evidence of photochromism with a colour change, from orange to red for **1** and from yellow to orange for **2** and **3**, upon irradiation with UV light.

## Conclusion   

The structures of three Schiff bases of salicyl­aldehyde derivatives with isoxazole derivatives, namely, (*E*)-2-{[(isoxazol-3-yl)imino]­meth­yl}phenol, **1**, (*E*)-2-{[(5-methyl­isoxazol-3-yl)imino]­meth­yl}phenol, **2**, and (*E*)-2,4-di-*tert*-butyl-6-{[(isoxazol-3-yl)imino]­meth­yl}phenol, **3**, are reported. The three structures all exist in the enol form and display an intra­molecular O—H⋯N hydrogen bond. All three structures contain inter­molecular C—H⋯N and C—H⋯O contacts. In the structures of **1** and **2**, π–π-type contacts were identified between the C=N group and the phenol ring. All three com­pounds had dihedral angles of <25°; however, none of the com­pounds were observed to be strongly thermochromic and even **1**, which was orange at room temperature, did not show a significant colour change upon cooling. This is in contrast to the anils where orange com­pounds with a dihedral angle of <25° are normally strongly thermochromic. All three title com­pounds did show evidence of photochromism upon irradiation with UV light.

## Supplementary Material

Crystal structure: contains datablock(s) 1, 2, 3, global. DOI: 10.1107/S2053229620010530/wv3001sup1.cif

Structure factors: contains datablock(s) 1. DOI: 10.1107/S2053229620010530/wv30011sup2.hkl

Structure factors: contains datablock(s) 2. DOI: 10.1107/S2053229620010530/wv30012sup3.hkl

Structure factors: contains datablock(s) 3. DOI: 10.1107/S2053229620010530/wv30013sup4.hkl

Characterization data for compounds 1-3. DOI: 10.1107/S2053229620010530/wv3001sup5.pdf

CCDC references: 2020495, 2020494, 2020493

## Figures and Tables

**Table 1:** Experimental details For all structures: *Z* = 4. Experiments were carried out with Mo *K*α radiation. H atoms were treated by a mixture of independent and constrained refinement.

	**1**	**2**	**3**
Crystal data			
Chemical formula	C_10_H_8_N_2_O_2_	C_11_H_10_N_2_O_2_	C_18_H_24_N_2_O_2_
*M*_r_	188.18	202.21	300.39
Crystal system, space group	Orthorhombic, *P*2_1_2_1_2_1_	Orthorhombic, *P* *n* *a*2_1_	Triclinic, *P* 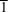
Temperature (K)	210	120	120
*a*, *b*, *c* (Å)	4.5999 (5), 10.2684 (10), 18.711 (2)	20.5584 (7), 10.0468 (4), 4.6417 (2)	10.8955 (5), 10.9571 (4), 14.8329 (6)
α, β, γ (°)	90, 90, 90	90, 90, 90	82.335 (3), 88.326 (4), 75.178 (3)
*V* (Å^3^)	883.79 (16)	958.73 (7)	1696.56 (12)
μ (mm^−1^)	0.10	0.10	0.08
Crystal size (mm)	0.3 × 0.08 × 0.05	0.49 × 0.24 × 0.09	0.6 × 0.31 × 0.18

Data collection			
Diffractometer	Bruker SMART APEXII area detector	Oxford Diffraction Xcalibur (Sapphire3, Gemini ultra)	Oxford Diffraction Xcalibur (Sapphire3, Gemini ultra)
Absorption correction	Multi-scan (*SADABS*; Bruker, 2012 ▸)	Analytical (*CrysAlis PRO*; Oxford Diffraction, 2010 ▸)	Multi-scan (*CrysAlis PRO*; Oxford Diffraction, 2010 ▸)
*T*_min_, *T*_max_	0.654, 0.746	0.969, 0.991	0.833, 1.000
No. of measured, independent and observed [*I* > 2σ(*I*)] reflections	10497, 2166, 1978	6756, 2021, 1819	14901, 6942, 5078
*R*_int_	0.020	0.040	0.037
(sin θ/λ)_max_ (Å^−1^)	0.667	0.641	0.625

Refinement			
*R*[*F* ^2^ > 2σ(*F* ^2^)], *wR*(*F* ^2^), *S*	0.032, 0.083, 1.08	0.037, 0.081, 1.05	0.049, 0.119, 1.02
No. of reflections	2166	2021	6942
No. of parameters	131	141	447
No. of restraints	0	1	0
Δρ_max_, Δρ_min_ (e Å^−3^)	0.18, −0.14	0.16, −0.17	0.26, −0.22

Computer programs: *APEX2* (Bruker, 2012 ▸), *CrysAlis PRO* (Oxford Diffraction, 2010 ▸), *SAINT* (Bruker, 2012 ▸), *SHELXT2018* (Sheldrick, 2015*a* ▸), *SHELXS97* (Sheldrick, 2008 ▸), *SHELXL2018* (Sheldrick, 2015*b* ▸) and *OLEX2* (Dolomanov *et al.*, 2009 ▸).

**Table 2:** Hydrogen-bond geometry (Å, °) for **1** [Chem scheme1]

*D*—H⋯*A*	*D*—H	H⋯*A*	*D*⋯*A*	*D*—H⋯*A*
O1—H1⋯N1	0.85 (3)	1.86 (3)	2.6110 (19)	146 (3)
C7—H7⋯N2^i^	0.93	2.71	3.599 (2)	159
C9—H9⋯O1^ii^	0.93	2.70	3.400 (2)	133
C9—H9⋯N2^i^	0.93	2.61	3.403 (2)	144
C10—H10⋯O1^i^	0.93	2.52	3.235 (2)	134

Symmetry codes: (i) 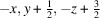; (ii) 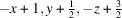.

**Table 3:** Hydrogen-bond geometry (Å, °) for **2** [Chem scheme1]

*D*—H⋯*A*	*D*—H	H⋯*A*	*D*⋯*A*	*D*—H⋯*A*
C7—H7⋯O2^i^	0.95	2.61	3.502 (3)	157
C7—H7⋯N2^i^	0.95	2.49	3.394 (3)	159
C9—H9⋯N2^i^	0.95	2.74	3.591 (3)	149
C2—H2⋯O1^ii^	0.95	2.62	3.496 (3)	153
O1—H1⋯N1	0.95 (3)	1.80 (3)	2.632 (2)	145 (3)

Symmetry codes: (i) 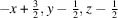; (ii) 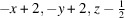.

**Table 4:** Hydrogen-bond geometry (Å, °) for **3** [Chem scheme1]

*D*—H⋯*A*	*D*—H	H⋯*A*	*D*⋯*A*	*D*—H⋯*A*
C23—H23⋯O2^i^	0.95	2.60	3.5232 (19)	165
C25—H25⋯N2^i^	0.95	2.70	3.637 (2)	169
C5—H5⋯O4^ii^	0.95	2.66	3.538 (2)	155
C7—H7⋯N4^ii^	0.95	2.82	3.708 (2)	156
C18—H18*B*⋯N2^iii^	0.98	2.67	3.559 (2)	152
O1—H1⋯N1	0.92 (2)	1.76 (2)	2.6207 (18)	153 (2)
O3—H3⋯N3	0.91 (3)	1.77 (2)	2.6062 (17)	151 (2)
C10—H10⋯O3^ii^	0.95	2.53	3.187 (2)	127
C28—H28⋯O1^i^	0.95	2.69	3.3370 (19)	126

Symmetry codes: (i) 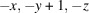; (ii) 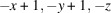; (iii) 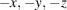.
