# Supplementary material for: Synthesis and structures of three isoxazole-containing Schiff bases
Source: Acta Crystallogr C Struct Chem. 2020 Aug 29;76(Pt 9):927–31. doi: 10.1107/S2053229620010530 (PMC7474185; doi:10.1107/S2053229620010530)
Supplement: Supplementary file 5 [file c-76-00927-sup5.pdf]

STRUCTURAL
CHEMISTRY

Volume 76 (2020)

Supporting information for article:

Synthesis and structures of three isoxazole-containing Schiff bases

Helen E. Mason, Judith A. K. Howard and Hazel A. Sparkes

S1. Characterisation

S1.1. (E)-2-((isoxazol-3-ylimino)methyl)phenol (1)

Compound **1** was obtained in a yield of 93% (0.875 g). *Elemental analysis, actual % (predicted %):* C = 62.02 (63.83); H = 4.29 (4.28); N = 15.62 (14.89). *Mass spectrometry [$C_{10}H_8N_2O_2$], actual m/z (predicted m/z):* ES+ spectrum shows one main $[M+H]^+$ peak; $[M+H]^+$ = 189.5 (189.2).

S1.2. (E)-2-(((5-methylisoxazol-3-yl)imino)methyl)phenol (2)

Compound **2** was obtained in a yield of 94% (0.947 g). *Elemental analysis, actual % (predicted %):* C = 64.70 (65.34); H = 4.98 (4.98); N = 13.82 (13.85). *Mass spectrometry [$C_{11}H_{10}N_2O_2$], actual m/z (predicted m/z):* ES+ spectrum shows one main $[M+H]^+$ peak; $[M+H]^+$ = 202.9 (203.2).

S1.3. (E)-2,4-di-tert-butyl-6-((isoxazol-3-ylimino)methyl)phenol (3)

Compound **3** was obtained in a yield of 94% (1.407 g). *Elemental analysis, actual % (predicted %):* C = 66.73 (71.97); H = 7.99 (8.06); N = 9.42 (9.33). *Mass spectrometry [$C_{18}H_{24}N_2O_2$], actual m/z (predicted m/z):* ES+ spectrum shows one main $[M+H]^+$ peak; $[M+H]^+$ = 301.3 (301.4).
